# Aligning the Aligners: Comparison of RNA Sequencing Data Alignment and Gene Expression Quantification Tools for Clinical Breast Cancer Research

**DOI:** 10.3390/jpm9020018

**Published:** 2019-04-03

**Authors:** Isaac D. Raplee, Alexei V. Evsikov, Caralina Marín de Evsikova

**Affiliations:** 1 Department of Molecular Medicine, Morsani College of Medicine, University of South Florida, Tampa, FL 33612, USA; iraplee@health.usf.edu; 2 Epigenetics & Functional Genomics Laboratory, Department of Research and Development, Bay Pines Veteran Administration Healthcare System, Bay Pines, FL 33744, USA; alexei.evsikov@va.gov

**Keywords:** atypia, breast neoplasms, ductal carcinoma in situ (DCIS), gene expression profiling, high-throughput nucleotide sequencing, infiltrating ductal carcinoma (IDC), paraffin embedding, sequence alignment, transcriptome

## Abstract

The rapid expansion of transcriptomics and affordability of next-generation sequencing (NGS) technologies generate rocketing amounts of gene expression data across biology and medicine, including cancer research. Concomitantly, many bioinformatics tools were developed to streamline gene expression and quantification. We tested the concordance of NGS RNA sequencing (RNA-seq) analysis outcomes between two predominant programs for read alignment, HISAT2, and STAR, and two most popular programs for quantifying gene expression in NGS experiments, edgeR and DESeq2, using RNA-seq data from breast cancer progression series, which include histologically confirmed normal, early neoplasia, ductal carcinoma in situ and infiltrating ductal carcinoma samples microdissected from formalin fixed, paraffin embedded (FFPE) breast tissue blocks. We identified significant differences in aligners’ performance: HISAT2 was prone to misalign reads to retrogene genomic loci, STAR generated more precise alignments, especially for early neoplasia samples. edgeR and DESeq2 produced similar lists of differentially expressed genes, with edgeR producing more conservative, though shorter, lists of genes. Gene Ontology (GO) enrichment analysis revealed no skewness in significant GO terms identified among differentially expressed genes by edgeR versus DESeq2. As transcriptomics of FFPE samples becomes a vanguard of precision medicine, choice of bioinformatics tools becomes critical for clinical research. Our results indicate that STAR and edgeR are well-suited tools for differential gene expression analysis from FFPE samples.

## 1. Introduction

After next-generation sequencing (NGS) technology was introduced in 2005, development of many high-throughput bioinformatics tools ensued, such as Bowtie, Tophat, Cufflinks, and CuffDiff “Tuxedo Suite” [1]. One of the most rapidly adopted NGS applications, RNA sequencing (RNA-seq), was introduced in 2008 and captures the transcriptome from cells or tissue samples. Bioinformatics tools have been developed in order to ease read mapping, splice junction, novel gene structure, and differential expression analysis of RNA-seq output. Sequence reads generated by RNA-seq can be assessed for single nucleotide polymorphisms (SNPs), splice variants, fusion genes, and individual transcript abundance in samples for differential expression analysis. Even in clinical settings, transcriptomics has been embraced for its potential for precision medicine in diagnoses, prognoses, and therapeutic decisions [2,3,4]. In comparison to the preceding popular technology for gene expression, microarrays, RNA-seq provides substantially increased transcriptome coverage and is more amenable to discovery science, as the identification of expressed loci or alternative splicing is not limited to the probes present on the array [5,6]. At the same time, gene expression analysis becomes computationally more challenging due to the requirement to correctly classify all sequence read outputs in RNA-seq datasets. As “wet lab” NGS technologies and “dry lab” high performance computers supporting NGS technology became more economical, RNA-seq and other NGS datasets expanded exponentially producing massive amounts of data [7]. Many researchers and clinicians, who utilize RNA-seq in their experiments, rely on outsourcing to cores or companies to generate and analyze their RNA-seq data. This practice is common with many niche experiments, especially in “omics”-based studies.

For an ideal RNA-seq experiment, researchers and clinicians would acquire freshly frozen tissue samples with minimal inclusion of non-target tissues, such as blood and fat, the most common contaminants. In reality, majority of clinical research relies on either archived tissue specimens, mainly as formalin-fixed, paraffin-embedded (FFPE) biopsies, which have increased RNA degradation and decreased poly(A) binding affinity [8,9,10], or punch biopsies for discrete tissue collection, which severely limits sample material [11]. We analyzed RNA-seq data from bore-dissected samples from FFPE breast tissue, which are known to be highly variable for quality and depth of sequencing results. These transcriptome datasets correspond to the typical quality that biomedical researchers will encounter using transcriptome studies for precision medicine, experiments, or re-analysis. The most common experimental question addressed in RNA-seq transcriptome studies is identifying differential expression among normal stages and stages of disease progression, and sometimes among treatment groups, to pinpoint pathways and putative molecular mechanisms underlying disease or its pathophysiology. To determine differential expression, RNA-seq reads need to be assessed for quality and then aligned to a reference genome. This underscores the essential importance to investigate the impacts of bioinformatics tools for sequence alignment and differential expression on accurate results and interpretation of transcriptome studies collected from FFPE specimens, which was the goal of our study. This paper also serves to point out the pragmatic shortcomings of universally applying a rigid “standard operating procedure” set of bioinformatics tools to RNA-seq data, by demonstrating the impacts and limitations of biological conditions, especially in sample processing and choice of programs.

We focused on two of the most popular sequence aligners, HISAT2 [12], and STAR [13], which superseded the once ubiquitous TopHat aligner program because of their superior computational speed. For differential expression, we used two differential gene expression testing tools, DESeq2 [14] and edgeR [15]. According to citation reports, currently edgeR and DESeq2 are the leading programs for RNA-seq data quantification and differential expression testing, with 10,013 and 8147 citations as of 22 February 2019 [16]. These popular bioinformatics tools are available for users via the open-source Galaxy platform [17], a portal designed to support a wide range of researchers from those with little or no experience or training, to professional bioinformaticians. We also investigated the strengths and weaknesses of the two most widely used differential expression analysis tools and present a straightforward bioinformatics pipeline from raw data to downstream analysis of gene expression, suitable even for researchers with minimal bioinformatics expertise. We performed differential gene expression analysis using the series of breast cancer progression RNA-seq data from micropunched FFPE samples and assessed similarities and differences in the results to detect the effects of different aligners upon read mapping affecting gene expression counts. Furthermore, we tested the impacts of different algorithms used to detect differential gene expression upon the list of statistically significant transcripts, as well as pathway analysis using Gene Ontology [18] enrichment.

## 2. Materials and Methods

### 2.1. Breast Cancer Samples

RNA-seq data used in this study are publicly available (BioProject ID: PRJNA205694 [19]). The dataset represents 72 RNA sequencing experiments from biopsies at different stages of breast cancer: 24 normal tissue samples, 25 early neoplasias (Atypia), 9 ductal carcinomas in situ (DCIS) and 14 infiltrating ductal carcinomas (IDC), from 25 patients. Briefly, RNA was extracted from core punches of FFPE specimens after histological confirmation of the cancer stage by board-certified pathologists and only samples that possessed >90% of luminal cells with the appropriate diagnosis were used for sequencing. Directional cDNA libraries were constructed and sequenced using Illumina GAIIx to obtain 36-base single-end reads.

### 2.2. RNA-seq Reads Alignment

We used two programs, STAR and HISAT2, which utilize different strategies to align the RNA-seq reads to human genome assembly. To improve alignment accuracy, both programs use a dataset of known splice sites to correctly identify potential spliced sequencing reads among RNA-seq data. This dataset, in “gene transfer format” (gtf), was obtained from ENSEMBL (release 87, 12/8/2016). Reads were aligned to the human reference genome assembly (hg19).

#### 2.2.1. STAR

STAR’s algorithm [13] uses a two-step approach. STAR aligns the first portion, referred to as “seed”, for a specific read sequence output against a reference genome to the maximum mappable length (MML) of the read. Next, STAR aligns the remaining portion, called the “second seed”, to its MML. After the read is completely aligned, STAR joins the two or more “seeds” together and scores the aligned reads based on a user-defined penalty for mismatches, insertions, and deletions. The joined seeds with the highest score are chosen as the correct alignment for a specific read sequence output. This approach allows for quick and easy annotation of multi-mapping reads with their own alignment scores. In our analysis, STAR was used with the following parameters:

*–seedSearchStartLmax 50 –seedSearchStartLmaxOverLread 1.0 –seedSearchLmax 0 –seedMultimapNmax 10000 –seedPerReadMax 1000 –seedPerWindowMax 50 –seedNonoLociPerWindow 10 –alignIntronMin 21 –alignIntronMax 0 –alignMatesGapMax 0 –alignSJoverhangMin 5 –alignSJDBoverhangmin 3 –alignSpliceMateMapLmin 0–alignSplicedMateMapeLminOverLmate 0.66 –alignWindowsPerReadNmax 10000 –alignTranscriptsPerWindowNmax 100 –alignTranscriptsPerReadNmax 10000 –alignEndsType Local*

#### 2.2.2. HISAT2

HISAT2 uses the Bowtie2 [20] algorithm to construct and search Ferragina–Manzini (FM) indices [21]. HISAT2 employs two types of indices for aligning: firstly, a whole-genome FM index to anchor alignments and secondly, numerous overlapping local FM indices for alignment extension. HISAT was used for our analysis with the following parameters:

*–mp MX=6, MN=2 –sp MX=2,MN=1 –np 1 –rdg 5,3 –rfg 5,3 –score-min L,0,-0.2 –pen-cansplice 0 –pen-noncansplice 12 –pen-canintronlen G,-8,1 –pen-noncanintronlen G,-8,1 –min-intronlen 20–max-intronlen 500000*

### 2.3. Gene Expression Counts

The simplest method for estimating transcript expression is to count the raw reads for each annotated gene locus in the genome assembly. This approach uses a gtf file containing genomic coordinates, such as gene nomenclature, positions in the chromosome for each exon, transcription start site, transcription termination site, etc. We used FeatureCounts [22], to extract information from the binary format for storing sequencing data, i.e., BAM files reads overlapping with genomic features in an input gtf file containing exon coordinates for all transcripts in the genome assembly using the following parameters: *–t ‘exon’ –g ‘gene_id’ –M –fraction –Q 12 –minOverlap 30*.

### 2.4. Data Normalization and Quality Control

#### 2.4.1. Data Normalization

To account for the depth of sequencing impacts, which affect read numbers of individual transcripts, we use normalized expression data, specifically counts per million (CPM) [23], to perform quality comparison of datasets. To calculate CPM values, we used the following formula:

*CPM_i_* = *R_i_*/*T_Ra_* × 1,000,000 (1)

where *CPM_i_* is a CPM value of a gene in a biological replicate; *R_i_* is the number of reads mapping to all exons of this gene in this biological replicate; *T_Ra_* is the total number of reads aligned (anywhere in the genome) from this biological replicate (i.e., the number of aligned reads in either STAR or HISAT2 output “binary alignment map” bam files). This procedure also transforms data from counts to a continuous scale.

#### 2.4.2. Quality Control

For quality control, we used ClustVis [24], a statistical tool for clustering of complex data such as RNAseq, based on principal component analysis and visualization of results. Any samples that fell outside of the initial 95% confidence interval on the two-dimensional PCA plot were flagged as outliers and removed before further analysis. ClustVis [24] has an intuitive user interface and was built using several R software packages to provide Principal Component Analysis and heat map plots of high-dimensional data from a data matrix. Data may be uploaded as text file, or manually entered into ClustVis text box. Rows (e.g., genes) and columns (e.g., samples) may contain multiple annotations to be detected automatically, or input manually, to provide additional features (e.g., color for groups, confidence intervals) applied to the PCA and heat map plots. 

### 2.5. Differential Gene Expression Analysis

Both tools are R packages and require raw read counts in a data matrix, which are normalized to account for differences in sequencing depth, and low count variability. Both tools assume RNA-seq data display overdispersion with variance greater than expected for random sampling. Both programs also assume RNA-seq data conform to a negative binomial distribution and employ Bayesian methods to fit raw gene expression counts into this distribution. To display differential expression outputs uniformly, we used the R software package Visualization of Differential Gene Expression using R (ViDGER) [25].

#### 2.5.1. DESeq2

This program, DESeq2, was used to detect differential expression in RNA-seq data. DESeq2 normalizes the counts of each gene employing a generalized linear model [26]. Afterwards, DESeq2 uses an empirical Bayes shrinkage to detect and correct for dispersion and log_2_-fold change (LFC) estimates.

#### 2.5.2. edgeR

The other popular program to detect differential expression, edgeR, uses a default method of normalization called trimmed mean of M-values, (TMM), which is obtained with the function: calcNormFactors. This method of normalization estimates the ratio of RNA production through a weighted trimmed mean of the log expression ratios. There are alternative normalization methods available in edgeR to account for data that fail to conform to a negative binomial distribution, which is assumed with TMM. To control for false discovery rate (FDR) we applied the estimateDisp function.

### 2.6. Gene Enrichment Analysis Using Visual Annotation Display (VLAD)

VLAD [27], accessible via Mouse Genome Informatics (MGI) web portal, is a powerful tool to find common functional themes in the lists of genes by analyzing statistical over- or underrepresentation of ontological annotations. Currently, users can choose among Gene Ontology (GO) [18] annotations for human genes, Gene Ontology, and Mammalian Phenotype Ontology (MP) [28] annotations for mouse genes, or upload a file of own annotations (in open biomedical ontology [29] “obo” format). Unlike other packages for ontological enrichment, VLAD uniquely allows simultaneous analysis and visualization of more than one query (i.e., several lists of genes may be analyzed and visualized simultaneously), as well as permits a user to provide own “universe set”, i.e., gene list to test queries.

## 3. Results

### 3.1. Bioinformatics Pipeline and Quality Control

**Figure 1:**
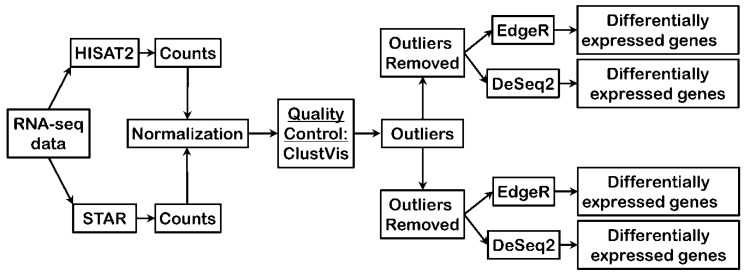
Bioinformatics pipeline employed to test differences in aligners and differential expression programs. RNA-seq: RNA sequencing.

RNA-seq data were analyzed using two different read aligners, STAR and HISAT2, to compare potential impacts of read mapping to the genome assembly upon the ultimate outputs of differential gene expression. Briefly, RNA-seq output reads were aligned to the genome assembly (hg19) by either HISAT2 or STAR, reads mapped to genes were counted for each individual gene to yield raw counts, subsequently count data were normalized, assessed for quality control using Principal Component Analysis using ClustVis, and sample outliers removed before performing differential gene expression analysis by two different programs, edgeR and DESeq2, to identify significant gene expression changes across breast cancer stages (Figure 1).

**Figure 2:**
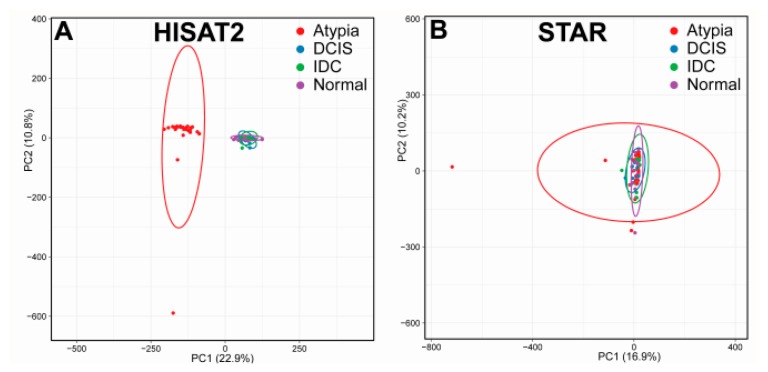
Principal Component Analysis (PCA) visualization of gene expression data from HISAT2 and STAR alignments. Clustering of samples on HISAT2 (**A**) and STAR (**B**) on the first two principal components before outlier removal.

To eliminate sample outlier biases, we performed Principal Component Analysis (PCA) of gene expression counts for each sample by each stage for both aligners. Gene expression counts were collected using featureCounts and normalized to the total number of aligned reads for each sample, and PCA completed on these data using ClustVis large edition (Figure 2). For all subsequent analysis, any samples that fell outside the 95% confidence ellipse in their respective stages (Normal, Atypia, DCIS, and IDC) were removed. For both HISAT2 and STAR, the same samples fell outside of the 95% confidence ellipse in each stage. In total, we identified six outlier samples in the RNA-seq dataset, which were: SRX286949 (normal tissue), SRX286945 and SRX286964 (atypia), SRX286961 (DCIS), and SRX286951 (IDC). Overall, Atypia stage presented more heterogeneity than any other stage, irrespective of the aligner. Unexpectedly, the PCA plots for all stages in HISAT2 data clustered atypia stage samples separately from all other stages (Figure 2A), which didn’t occur in PCA plots for STAR data (Figure 2B).

### 3.2. Outputs of Aligners

**Figure 3:**
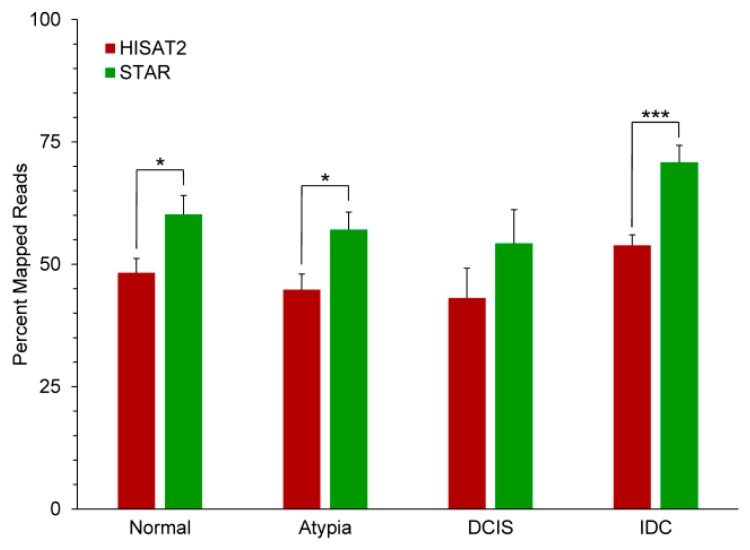
Performance of HISAT2 and STAR aligners on the breast cancer series data. * *p* < 0.05, *** *p* < 0.001 2-tailed *t*-tests between aligners at each stage.

All reads for all samples were aligned to the human genome assembly (hg19). Overall, STAR significantly outperformed HISAT2 in aligning the FASTQ reads to the genome (Figure 3). The generally low proportion of aligned reads to all input reads for both programs is likely due to the quality of the libraries, as a significant number of input reads were poly(A) sequences, Illumina adapter sequences, and reads corresponding to the very 3′-ends of mRNAs, which are too uninformative for correct mapping (Supplemental Table S1).

### 3.3. Gene Expression Profiling

#### 3.3.1. Highly Expressed Genes

**Figure 4:**
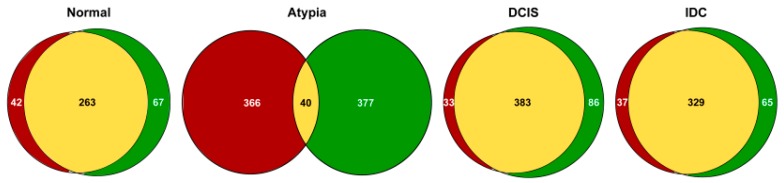
Overlap between the highest expressed genes in the breast cancer datasets aligned by HISAT2 or STAR. HISAT2-identified genes are in red; STAR genes are in green; overlapping genes are in yellow.

To determine how concordant the alignment tools were in mapping the reads to the genome, we compared the highest expressed genes that correspond to 50% of all reads mapped to exons. In the normal samples 50% of the mapped reads came from 330 and 305 genes for STAR and HISAT2 respectively and they shared 263 of those genes (Figure 4). In atypia samples, 50% of the mapped reads came from 417 and 406 genes for STAR and HISAT2 respectively and they shared only 40 of those genes. In DCIS samples, 50% of the exon-mapped reads came from 469 and 416 genes for STAR and HISAT2, respectively; of those, 383 genes were shared. In IDC samples, 50% of the exon-mapped reads came from 384 and 366 genes for STAR and HISAT2, respectively, and the lists shared 319 of those genes. The high amount of discrepancies in atypia convinced us to investigate the underlying factors resulting in the major differences in alignment.

#### 3.3.2. Alignment to Pseudogenes

**Figure 5:**
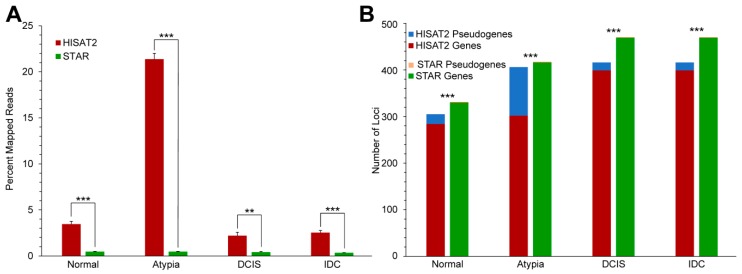
Expression of pseudogenes in HISAT2 and STAR alignment data. (**A**) Percent of all reads, by stage, aligned to annotated pseudogene loci by HISAT2 (red) and STAR (green). (**B**) Number of retrogenes among highest-expressed genes by stage and aligner. ** *p* < 0.01, *** *p* < 0.001, 2-tailed *t*-tests (**5A**), or *χ^2^* tests (**5B**). DCIS: ductal carcinoma in situ; IDC: infiltrating ductal carcinoma.

Processed pseudogenes, sometimes referred to as non-functional retrogenes, are intronless gene copies produced by reverse transcription of an original “parent” gene mRNA and insertion of the resulting cDNA copy elsewhere in the genome. Processed pseudogenes are non-functional and comprise the bulk of the known loci in the broad category of “pseudogenes”, i.e., genomic loci harboring similarity to a protein-coding gene but not having any recognized biological function. The sequence similarity among processed pseudogenes and their parent genes poses a problem for aligners, whose algorithms have to decide when assigning a read to a particular locus in the genome. To determine the underlying factors causing the different alignments, we analyzed the numbers of reads mapped to pseudogenes by HISAT2 and STAR. Between the two aligners tested, HISAT2 consistently had significantly higher amounts of reads aligned to pseudogenes when compared to STAR (Figure 5A). Furthermore, for Atypia stage, HISAT2 had drastically higher amounts of reads aligned to pseudogenes than the other stages. To determine what portion of the top 50% of mapped reads were pseudogenes, we obtained a list of pseudogenes from the hg19 gtf annotation file we used and compared this list with the top 50% of mapped genes for each stage and each aligner. A single pseudogene was in the gene list for each stage which represented the top 50% of mapped reads for STAR. Conversely, HISAT2 consistently had higher amounts of pseudogenes represented in the top 50% of mapped reads (Figure 5B).

### 3.4. Differential Gene Expression Analysis

The differential expression comparison on data from different alignment tools was done to further explore the consequences of previously described alignment tool biases, as well as to compare the two popular tools used for the purpose of identification and quantification of gene expression differences between conditions, edgeR and DESeq2.

#### 3.4.1. edgeR Following HISAT2 or STAR

**Figure 6:**
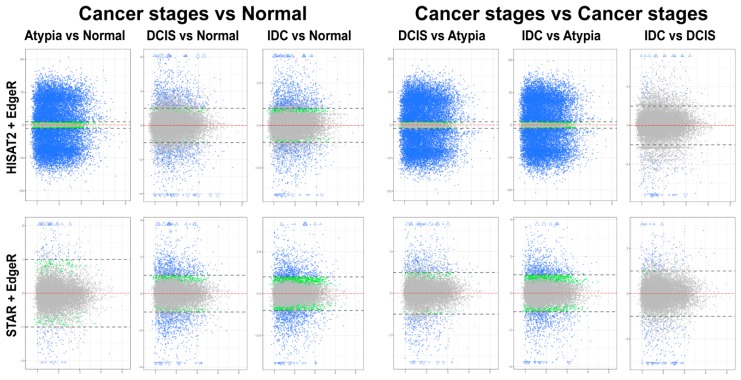
Mean-average (MA) plots of pairwise comparisons of all stages using edgeR. HISAT2 (top) and STAR (bottom) gene counts to identify differentially expressed genes. Each gene is represented by a single dot. Blue dots represent genes with significant expression difference and LFC ≥ 1. Blue triangles represent genes with significant expression difference and LFC is off scale. Green dots represent genes with significant expression difference but LFC < 1; grey dots represent statistically non-significant genes. Y-axis (all plots): log_2_ of expression fold change; X-axis (all plots): log_2_ of mean value of gene expression across all stages.

Overall patterns of differential gene expression performed by edgeR on HISAT2 or STAR data were similar for all pairwise stage comparisons, except atypia versus any other stage (Figure 6). HISAT2 consistently had the atypia stage comparisons produce >15,000 statistically significant differentially expressed transcripts with log_2_ fold change LFC ≥1 or LFC ≤−1 (i.e., at least two-fold increase or decrease in transcript expression levels between stages) (Figure 6, top panels). Conversely, differential expression pairwise comparisons with STAR atypia stage versus each other stage identified 350 to 2496 differentially expressed genes (Figure 6, bottom panels).

#### 3.4.2. DESeq2 Following HISAT2 and STAR

**Figure 7:**
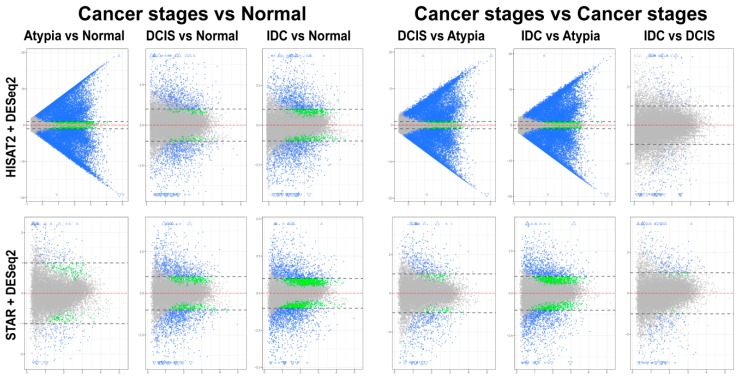
Mean-average (MA) plots of pairwise comparisons of all stages using DeSeq2. HISAT2 (top) and STAR (bottom) gene counts for all samples were analyzed to identify differentially expressed genes. Each gene is represented by a single dot. Blue dots represent genes with significant expression difference and log2-fold change (LFC) ≥ 1. Blue triangles represent genes with significant expression difference and LFC is off scale. Green dots represent genes with significant expression difference but LFC < 1; grey dots represent statistically non-significant genes. Y-axis (all plots): log_2_ of expression fold change; X-axis (all plots): log_2_ of mean value of gene expression across all stages.

Differential expression analysis using DESeq2 on pairwise comparisons of STAR alignments revealed 255 transcripts having LFC > 1 and 177 genes with LFC < 1 in normal versus atypia comparisons. Normal versus DCIS and Normal versus IDC analysis revealed 1677 LFC > 1, 482 LFC < 1, and 2304 LFC > 1, 1417 LFC < 1 differentially expressed genes, respectively (Figure 7, bottom panels). Similarly to edgeR, differential expression analysis of HISAT2 with DESeq2 consistently produced high numbers of statistically significant differentially expressed genes in atypia pairwise comparisons, >19,000 genes with LFC > 1 (Figure 7, top panels). Normal versus Atypia, Normal versus DCIS, and Normal versus IDC analysis revealed 19,419 LFC > 1, 1212 LFC < 1, 1585 LFC > 1, 190 LFC < 1, and 2196 LFC > 1, 732 LFC < 1 differentially expressed genes, respectively.

#### 3.4.3. Comparison of DESeq2 and edgeR Results

**Figure 8:**
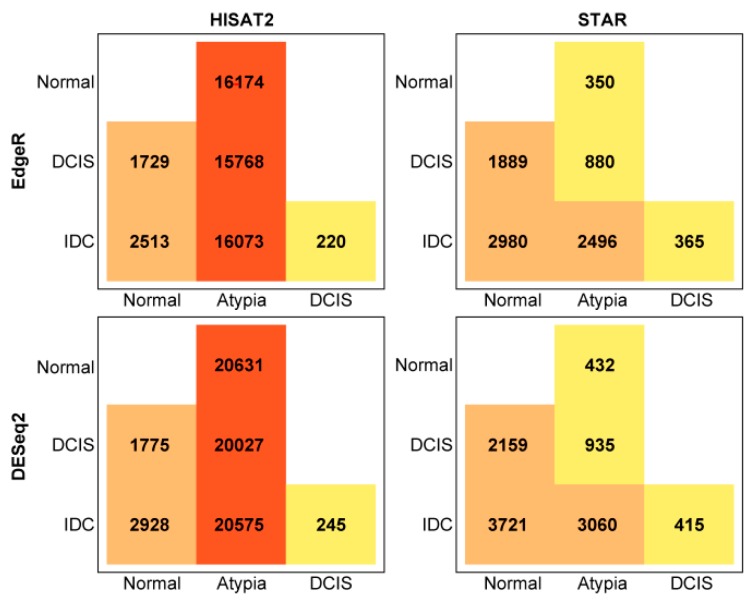
Total number of differentially expressed genes in pairwise comparisons, by aligner (HISAT2 or STAR), and quantification program (edgeR or DESeq2).

The total number of statistically significant differentially expressed genes in pairwise comparisons, by aligner and differential expression tool, is summarized in Figure 8. Overall, DESeq2 produced more inflated lists comparing to edgeR.

**Figure 9:**
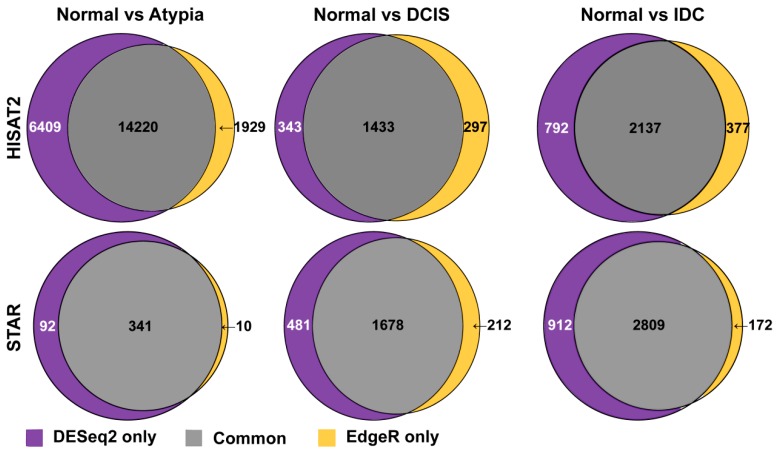
Overlap among genes identified as differentially expressed by either DESeq2 or edgeR in HISAT2 or STAR-aligned RNA-seq data.

Next, to compare how similar these most common differential gene expression analysis tools, edgeR and DESeq2, calculate expression differences on the same data, we compared lists of all differentially expressed genes generated by these programs and produced Venn diagrams, for each pairwise cancer stage comparison to normal samples. DESeq2 and edgeR shared 14,220, 1433, and 2137 of the differentially expressed genes on the HISAT2 alignment pairwise comparisons for Normal versus Atypia, Normal versus DCIS, and Normal versus IDC, respectively (Figure 9, top row). Notably, at the Atypia stage, HISAT2 yielded a disproportionally high amount of differentially expressed genes detected by both edgeR and DESeq2 programs (14,220 common genes, 6409 DESeq2 only, and 1929 edgeR only; Figure 9 top left panel), reflecting the global RNA-seq data misalignment to processed pseudogenes by HISAT2 (Figure 4, Figure 5, Figure 6 and Figure 7). DESeq2 and edgeR shared 341, 1678, and 2809 of the differentially expressed transcripts on the STAR alignment pairwise comparisons for Normal versus Atypia, Normal versus DCIS, and Normal versus IDC, respectively (Figure 9, bottom row). Overall, STAR alignment yielded the highest percent of overlapping genes between differential expression programs.

#### 3.4.4. Downstream Analysis of Gene Expression

**Figure 10:**
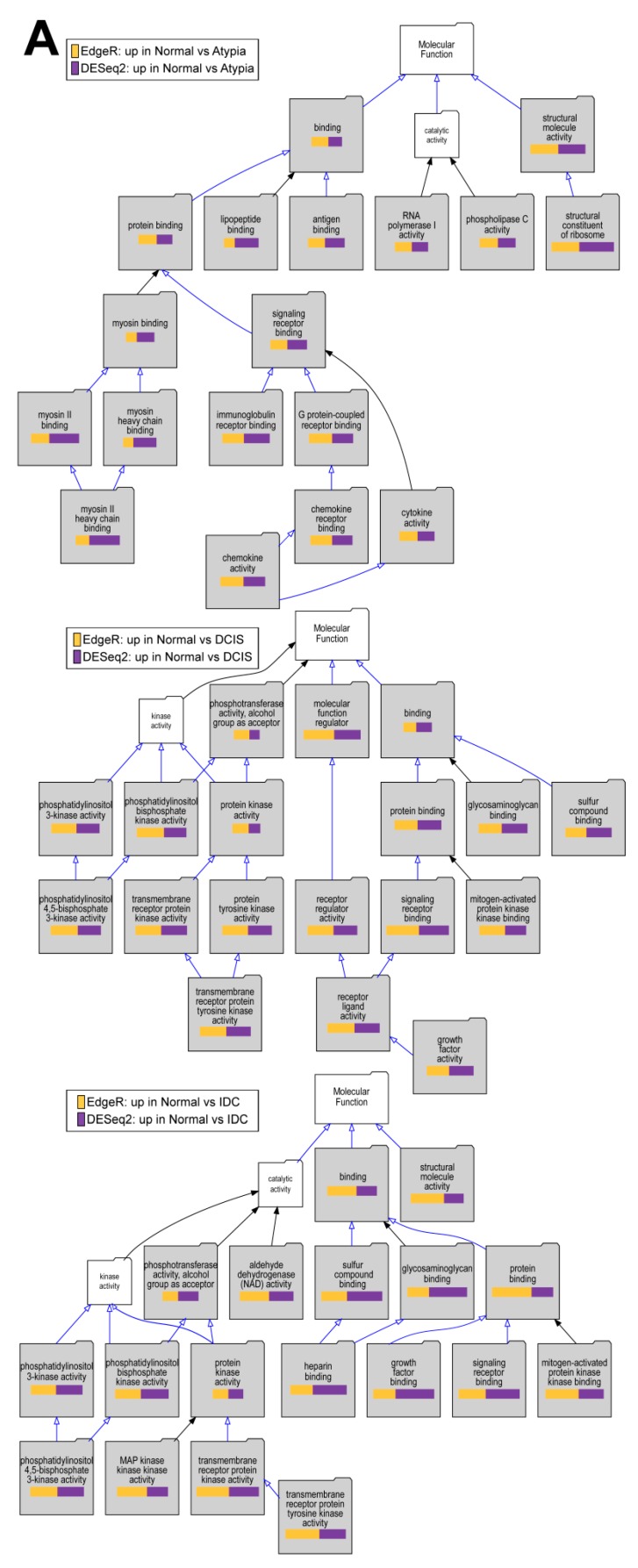

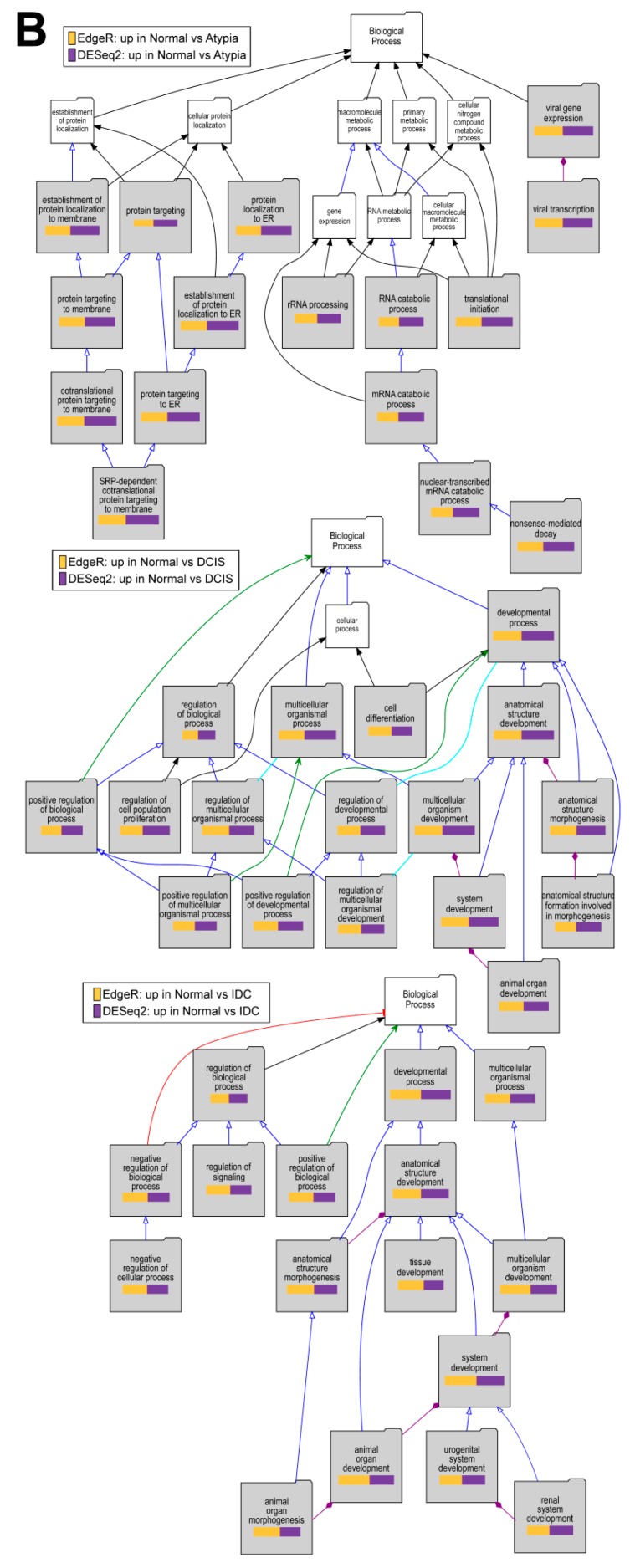

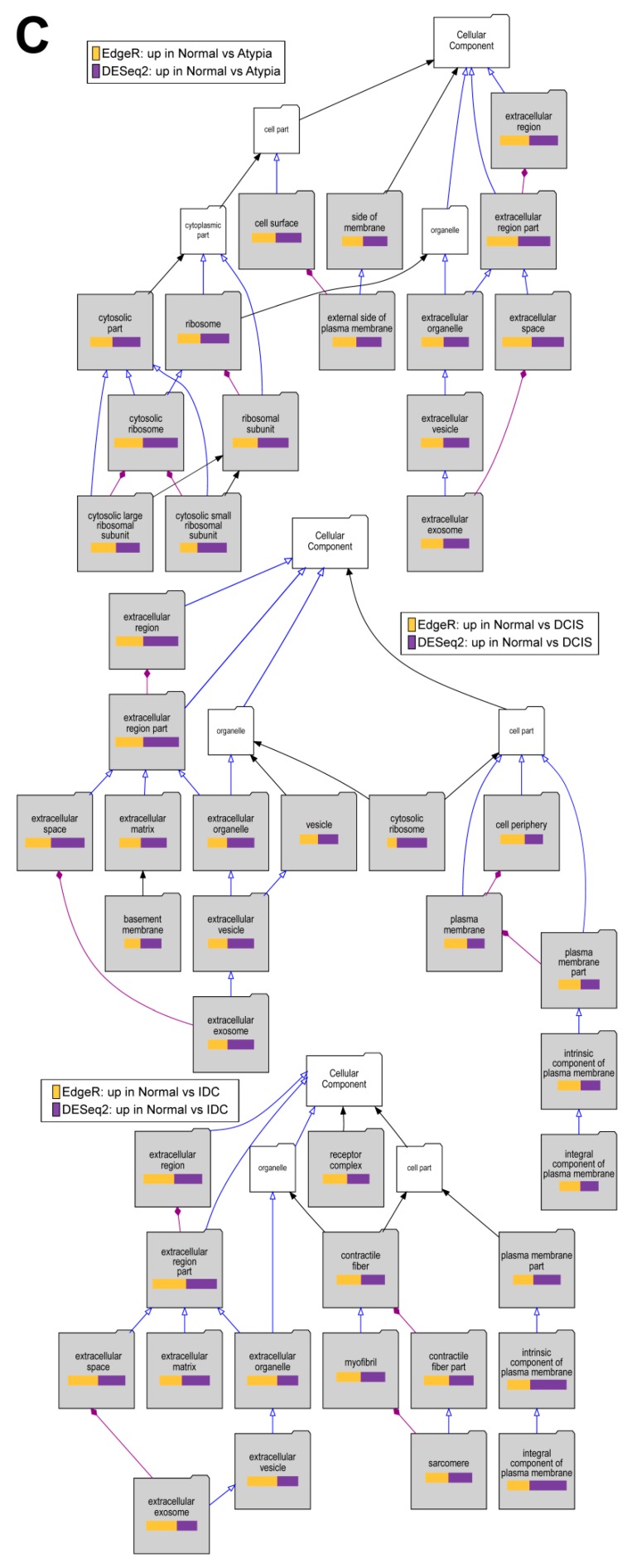
Overrepresented Gene Ontology terms among genes upregulated in normal samples comparing to cancer stages. (**A**) Molecular Function, (**B**) Biological Process, and (**C**) Cellular Component Gene Ontology (GO) categories; Top to bottom: Atypia versus Normal, DCIS versus Normal and IDC versus normal comparisons. Significant GO terms are shaded; Size of the bar represents significance of *p*-value, and *p*-value ratios between edgeR and Deseq2.

To test if downstream analysis of differentially expressed genes is affected by the type of software used to identify these genes, we performed Gene Ontology enrichment studies with the Visual Annotation Display tool (VLAD) for genes found significantly downregulated in pairwise comparisons of cancer stages (Atypia, DCIS, IDC) to normal samples by each program (DESEq2, edgeR), using gene expression data only from STAR alignments because of the global misalignment to pseudogenes by HISAT2. The pathways represented in the GO category Molecular Function, with data from expression significantly decreased in DCIS or IDC compared to normal samples, indicate phosphatidylinositol kinase pathways (Figure 10A), which corroborate the previously published work on this dataset. Our analysis extends these previous findings because we provide evidence of fundamental developmental pathways being decreased across all stages compared to normal samples (Figure 10B). In addition, significant decrease of extracellular matrix genes, such as basement membrane, was found in cancer stages comparing to normal (Figure 10C). Overall, there was high concordance among overrepresented Gene Ontology terms identified among either DESeq2 or edgeR-identified genes significantly downregulated in cancer (Supplemental Table S2). Similar concordance in significantly enriched GO terms between DESeq2 or edgeR-identified genes was observed for genes upregulated in cancer (Supplemental Table S3).

## 4. Discussion

Personalized medicine is a data science approach that promises focused treatments based on an individual’s sequenced genome to precisely target pathways underlying disease or its symptoms. To date only a few genotypes are robustly identified in breast cancer [30], such as *BRCA1* and *BRCA2*, which is inadequate to achieve the full promise of personalized medicine. A promising alternative to this genetic approach to personalized medicine is transcriptome-based identification of altered pathways of the diseased tissue to target its underlying disrupted cellular and molecular machinery. Identification of affected pathways using bioinformatics pipelines for RNA-seq data empowers clinicians to make a focused and informed decision on specific altered genes and pathways as potential therapeutic targets in precision medicine for individual patients. The goal of our studies was to unveil potential hidden biases arising from different bioinformatics analysis pipelines, and to find practical solutions to circumvent and mitigate these biases that impact the downstream analyses in transcriptomics experiments. Our results indicate that STAR and edgeR are robust and well-suited bioinformatics tools for transcriptomics pipelines of the most commonly used clinical specimens, biopsies from FFPE tissues, to precisely and accurately align and detect differential gene expression.

After initial quality control checks on the raw output from the sequencer, alignment is the first step in RNA-seq analysis and all subsequent analysis relies profoundly upon this initial step [31]. Typically, reads obtained from sequencing will be mapped and aligned to a reference genome, which is particularly prone to errors for RNA-seq data because of the presence of output reads spanning exon-exon splice junctions. The most common software platforms available for mapping to a reference genome, TopHat [32], HISAT2 [12], and STAR [13], identify splice junctions. These platforms differ in computational speed and memory usage, and in their algorithms for handling base and splice junction alignment precision. TopHat is currently becoming obsolete and has been superseded by HISAT2 due to relative computational inefficiency. TopHat and HISAT2 are built on the short read mapping program Bowtie2 [20]. While all three aligners are considered fast, HISAT2 and STAR consistently outperform TopHat with respect to computational speed [13,33,34]. Although all three aligners performed well in aligning a read onto the respective genomic locus, notable discrepancies and deficiencies were found for TopHat yielding insufficient genomic mapping for reliable downstream analysis. Given these reasons, and the fact that TopHat performance has been evaluated previous [34,35], our studies assessed the alignment performance between STAR and HISAT2.

The next step in bioinformatics pipelines, quantification of gene expression, is commonly performed with DESeq2 or edgeR programs [14,15]. A common drawback during this step is that difference in sequencing depth between samples or groups by itself can skew results when estimating differences in gene expression levels. The relative expression level of genes is often estimated based on the number of mapped reads. These counts are subjected to statistical tools to assess significant differences between groups. However, there is much confusion in the literature when reporting relative expression level units in RNA-seq data. The confusion stems from the different forms of normalization required for within versus between sample comparisons. Many methods for within sample comparison attempt to correct for sequencing depth and gene length. These methods produce the most frequently reported unit of expressions for RNA-seq data, which are read per kilobase of exon per million reads (RPKM), fragments per kilobase of exon per million of reads (FPKM), and transcripts per million (TPM) [36]. The order in which RPKM and FPKM normalize the read counts causes differences within samples that should not be ignored. Instead, when comparing within samples one should use TPM values which eliminates the invariance [36]. A relationship among RPKM, FPKM, and TPM is further discussed elsewhere [37]. In this study, to account for the impact of sequencing depth, we normalized expression data to counts per million (CPM) [23], which equal TPM values in single-end sequencing RNA-seq datasets, to perform quality comparison between the sample datasets. This normalization has a number of advantages for FFPE samples, where RNA quality is low, captured cDNA is not sheared, and there is no need to control for transcript length. Additional advantage of normalization is conversion of gene expression data from counts to a continuous scale.

**Figure 11:**
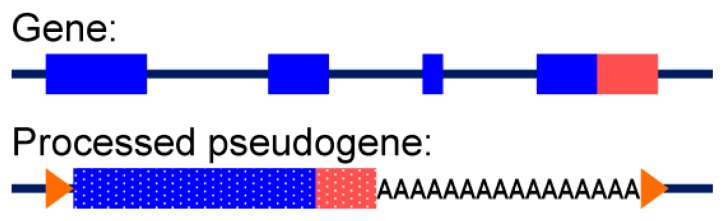
Schematic representation of a gene versus its processed pseudogene. Unlike genes, processed pseudogenes lack introns and have a poly (A) stretch at the 3′ end. Blue represents a coding part of the sequence, and red represents a 3′ untranslated region (UTR). Orange arrowheads represent target site duplications (TSDs), a feature of processed pseudogene insertions.

In our study, STAR had the highest average rates of mapped reads for each stage, whereas HISAT2 aligned fewer reads and importantly, had increased rates of alignment to pseudogenes instead of genes, which clearly compromised alignment fidelity, leading to skewed gene expression counts and likely erroneous outputs with either edgeR or DESseq2 programs, albeit the latter produced more expanded lists of differentially expressed genes. For these FFPE specimens, STAR alignment yielded more precise and accurate results with the fewest misalignments to pseudogenes compared to HISAT2. We speculate the preferential alignment to pseudogene regions of the genome is caused by the innate bias from 3′-end mRNA enriched libraries derived from FFPE samples contain a large proportion of reads corresponding to 3′ ends of mRNAs, which span into the poly(A) tails. Consequently, these reads better match to processed pseudogenes, which contain poly(A) stretches absent in genes (Figure 11). Indeed, alignment of these reads to a processed pseudogene will yield a higher score compared to its actual gene, resulting in incorrect alignment by the algorithm. STAR algorithm trims the poly(A) sequences from the reads [13] to counter this common library bias. Therefore, differences between aligners will be most pronounced for the FFPE specimen-derived RNA-seq libraries, or any other libraries from substantially degraded and fragmented mRNA that were produced with oligo(dT) priming, because these libraries are extremely enriched with 3′-end reads. In cases when libraries are produced using high-quality RNA samples with oligo(dT) priming, such differences will be less visible because a substantially smaller portion of reads would correspond to the 3′UTR-poly(A) junction in mRNA.

Our results clearly demonstrate alignment impacts and play a large role in bioinformatics analysis outcomes, especially to detect and identify differential gene expression. Our data, together with other comparative tests of different programs [34,35], indicate a single aligner program cannot be applied universally to RNA-seq datasets. It is possible that short output nucleotide sequences may have contributed to the FM index generation utilized by HISAT2 with the annotated settings, propagating misalignments to pseudogenes. While notable improvements in sequencing technology have increased nucleotide output read length to greater than 300 nucleotides, which may increase alignment accuracy and mitigate misalignment to pseudogenes, Chhanawala and co-authors [38] reported that output reads >25 nucleotides had negligible impacts in detecting differential expression.

A recent study [39] compared the performance and accuracy of the most commonly used differential expression tools available for RNA-seq analysis and discovered DESeq2 and edgeR outperformed all other tools with the lowest false discovery rate (FDR) and highest true discovery rate (TDR). It appeared DESeq2 slightly outperformed edgeR with respect to FDR in datasets with large number (>12) of biological replicate samples, however we observed DESeq2 over-predicted differentially expressed genes. This differs from earlier reports of edgeR’s propensity to a higher FDR at higher number of biological replicates. The addition of the estimateDisp function may have applied heavier weighted likelihood empirical Bayes methods to obtain the posterior dispersion estimates [40]. Therefore, in studies with a large cohort of replicates, both DESeq2, and edgeR are recommended to be used with the estimateDisp function to control FDR. More commonly for biomedical research and personalized medicine, i.e., in studies with fewer than 12 replicates, edgeR has advantage in reducing false negative rates (FNR).

Our study clearly demonstrates the need for heedful intent and meticulous review executing a bioinformatics pipeline to assess differential expression of RNA-seq data for clinical diagnoses, prognosis, and choice of treatment. Bioinformaticians need to be aware of the biological and clinical impacts, and limitations, of the specimen collection and preservation techniques for samples used for RNA-seq when creating custom transcriptomic pipelines, especially employing an SOP “standard ‘omics pipeline” in a genomics core setting. Due to the increased use of RNA-seq to diagnose, prognose, and generate therapeutic options clinicians and biomedical researchers need to more closely obtain a stronger foundation and understanding of the bioinformatics tools and pipelines analyzing RNA-seq data and generating the results. This study highlights possible limitations of this version of HISAT2 for some RNA-seq read generation technologies, poor quality samples, and short RNA seq reads, thus providing clinicians with insights for choosing the right bioinformatics tools for the job. In cases when paired (i.e., within patients) analysis is possible, it can be a valuable tool when between-analysis produces no significant result. However, in the breast cancer progression dataset only 3 of 25 patients had all stages of breast lesions (Normal, Atypia, DCIS, and IDC) represented. Therefore, we used between analysis, which produces a more conservative statistical cutoff than a paired analysis. In our ClustVis quality control analysis, we found no evidence for batch effects in the analyzed dataset, albeit when batch effects are detected, additional modules can be used in edgeR, or adjust data model in DeSeq2, to extract significant gene expression differences. A recent review has an excellent discussion of possible interventions for batch effects when detected in RNA-seq data [41].

Application of transcriptomics can facilitate the exploration of underlying pathogenic mechanisms, identification of genetic variants, and determination of treatment effects, including screening for molecular biomarkers. Importantly, expression signatures in diseased phenotypes may pinpoint precise interventions required to alleviate the disease state, a goal of precision medicine, without a need for the cost-prohibitive “personalized” assembly and analysis of patient’s own genome. Thus, transcriptomics can classify individuals, while simultaneously facilitating discovery, testing, and validation of new therapeutics for breast cancer patients, defined at the cellular and molecular levels.

## 5. Conclusions

Transcriptomics is an effective tool for both diagnostics and discovery science, exposing novel cellular and molecular mechanisms in clinical and translational models to yield robust targets for drug discovery to identify and test novel therapeutics. The cost and time required for transcriptome analysis have been greatly reduced by the development of next generation sequencing. Our results underscore the essential importance to investigate the impacts of bioinformatics tools for sequence alignment and differential expression on accuracy of results and interpretation of transcriptome studies collected from FFPE specimens, which was the goal of our study. This paper also serves to point out the pragmatic shortcomings of universally applying a rigid “standard operating procedure” set of bioinformatics tools to RNA-seq data, by demonstrating the impacts and limitations of biological conditions, especially in sample processing and choice of programs.

## Supplementary Materials

The following are available online at https://www.mdpi.com/2075-4426/9/2/18/s1, Table S1: Overrepresented reads in RNA-seq datasets used in this study, Table S2: Statistically significant genes upregulated in normal samples comparing to cancer stages, Table S3: Statistically significant genes upregulated in cancer stages comparing to normal samples.
